# Climate Change Effects on Secondary Compounds of Forest Trees in the Northern Hemisphere

**DOI:** 10.3389/fpls.2018.01445

**Published:** 2018-10-02

**Authors:** Jarmo K. Holopainen, Virpi Virjamo, Rajendra P. Ghimire, James D. Blande, Riitta Julkunen-Tiitto, Minna Kivimäenpää

**Affiliations:** ^1^Department of Environmental and Biological Sciences, Kuopio Campus, University of Eastern Finland, Kuopio, Finland; ^2^Department of Environmental and Biological Sciences, Joensuu Campus, University of Eastern Finland, Joensuu, Finland

**Keywords:** CO_2_, drought, ozone, phenolics, temperature, terpenes, UV-B, VOCs

## Abstract

Plant secondary compounds (PSCs), also called secondary metabolites, have high chemical and structural diversity and appear as non-volatile or volatile compounds. These compounds may have evolved to have specific physiological and ecological functions in the adaptation of plants to their growth environment. PSCs are produced by several metabolic pathways and many PSCs are specific for a few plant genera or families. In forest ecosystems, full-grown trees constitute the majority of plant biomass and are thus capable of producing significant amounts of PSCs. We summarize older literature and review recent progress in understanding the effects of abiotic and biotic factors on PSC production of forest trees and PSC behavior in forest ecosystems. The roles of different PSCs under stress and their important role in protecting plants against abiotic and biotic factors are also discussed. There was strong evidence that major climate change factors, CO_2_ and warming, have contradictory effects on the main PSC groups. CO_2_ increases phenolic compounds in foliage, but limits terpenoids in foliage and emissions. Warming decreases phenolic compounds in foliage but increases terpenoids in foliage and emissions. Other abiotic stresses have more variable effects. PSCs may help trees to adapt to a changing climate and to pressure from current and invasive pests and pathogens. Indirect adaptation comes via the effects of PSCs on soil chemistry and nutrient cycling, the formation of cloud condensation nuclei from tree volatiles and by CO_2_ sequestration into PSCs in the wood of living and dead forest trees.

## Introduction

Plant secondary compounds (PSCs) have high chemical diversity with an estimated 200,000 compounds (Dixon and Strack, 2003). In higher plants, terpenoids (approximately 30,000 known compounds, Lämke and Unsicker, 2018), alkaloids (21,000) (Wink, 2010), and phenolic compounds (8,000) (Munné-Bosch, 2012) are the most diverse PSC groups. The majority of PSCs are related to plant chemical defense and herbivore pressure, which are considered the main drivers of PSC diversification (Lämke and Unsicker, 2018). Terpenoids originate directly from glycolysis of glucose via the mevalonate pathway or the methylerythritol phosphate (MEP) pathway (Bartram et al., 2006). Phenolic compounds originate from the shikimic acid pathway, which is related to the metabolism of carbohydrates and aromatic amino acids (Seigler, 1998; Lindroth, 2012). Most of the alkaloids are derived from amino acid precursors (Wink, 2010), but coniferous alkaloids are synthesized via the polyketide pathway (Seigler, 1998). Terpenes and phenolics are the most extensively studied PSCs in forest trees (Lindroth, 2012; Lämke and Unsicker, 2018), while alkaloids have been considered in relatively few studies (Virjamo et al., 2014). Alkaloids and phenolics may compete more with protein synthesis than terpenoids (Koricheva, 2002). Up to 10% of recently fixed carbon can be allocated to volatile organic compounds (VOCs) in stressed plants (Peñuelas and Staudt, 2010), while stored terpenoids typically require secretory structures such as resin canals and glandular trichomes to avoid autotoxicity (Goodger et al., 2013).

At the global level, climate change (CC) is strongly linked to increasing CO_2_ concentration in the atmosphere, which affects temperature globally (IPCC, 2014). Depletion of the stratospheric ozone (O_3_) layer is partly affected by elevated CO_2_ and leads to increased UV-B radiation (IPCC, 2014). In the lower atmosphere (troposphere) O_3_ acts as a greenhouse gas and is phytotoxic to plants (Vapaavuori et al., 2009; Lindroth, 2010). At the regional scale, global warming affects arctic and boreal areas most strongly and will include drastic changes, e.g., in precipitation, drought, and cloudiness (shading), affecting temperature-dependent ecosystem processes in forests such as nutrient availability (IPCC, 2014).

**FIGURE 1:**
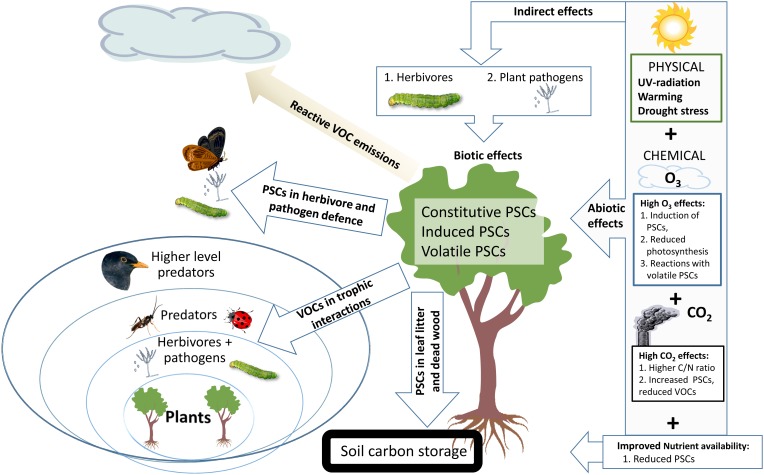
Global climate change – related abiotic and biotic stresses and their influence on types of plant secondary compounds (PSCs) in forest trees. Ecosystem level feedbacks transmitted by leaf PSCs are indicated with arrows on the left from the target tree. PSCs in foliage provide chemical defenses against herbivores and pathogens (Lämke and Unsicker, 2018). PSCs of leaf and needle litter affect mostly on tree nutrient uptake (Smolander et al., 2012) and rhizosphere organisms while PSCs in deadwood are part of important carbon storage (Pan et al., 2011) and stored PSCs could mitigate wood decay by decomposer organisms (Nerg et al., 2004; Karppanen et al., 2007) and release of CO_2_ to the atmosphere. Volatile PSCs (VOCs) affect the trophic interactions in the forest ecosystem where the tree is growing (Blande et al., 2014), while reactive VOCs affect the atmosphere and may have atmosphere-biosphere level feedbacks in the surrounding ecosystems (Joutsensaari et al., 2015).

Climate change directly affects the abiotic conditions of forest trees and their growth, physiology and defense including induced PSC production (Metlen et al., 2009; **Figure 1**). The responses of other organisms, such as microbial plant pathogens, herbivorous insects and arrival of exotic invasive herbivores and pathogens will alter the biotic stress burden of trees (Donnelly et al., 2012). The richness of chemical compounds originating from PSCs in forest ecosystems is affected by PSC reactivity (Kundu et al., 2012). Many of the volatile reaction products form secondary organic aerosols (SOA), which may affect cloud formation (Zhao et al., 2017; Rose et al., 2018; Scott et al., 2018) and provide important ecosystem – atmosphere feedback, thus helping vegetation to adapt to CC (e.g., Niinemets, 2018). SOA particles may become wet or dry deposited on forest vegetation and accumulate in soil (Holopainen et al., 2017) together with litter PSCs (Smolander et al., 2012).

**FIGURE 2:**
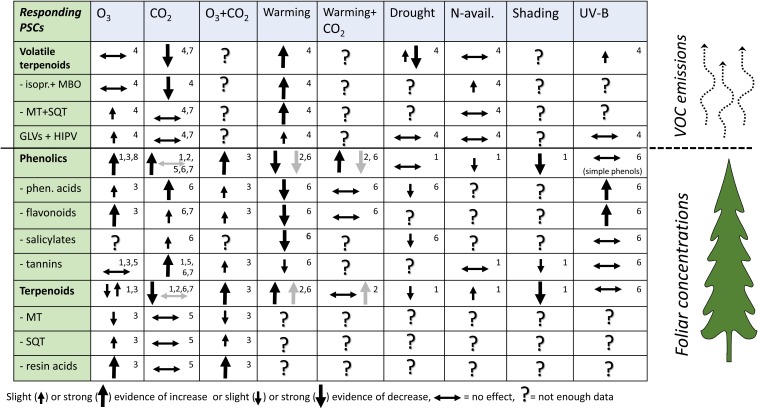
Summary of the results in comprehensive reviews and meta-analyses of plant PSCs responses in their concentration or emissions in forest trees under climate change related stresses. This tabulation covers nearly 400 original scientific articles. Type of stresses: O_3_ = elevated ozone, CO_2_ = elevated carbon dioxide, UV-B = elevated UV-B radiation. The tabulation gives the direction of PSC responses to single environmental factors, except for the combined O_3_+CO_2_ and CO_2_+warming effects. Arrow direction (increase or decrease) shows the effect size (significant or highly significant effect in meta-analysis) or the amount of evidence in reviews. Horizontal bidirectional arrow indicates the studies with non-significant results and up and down arrows in the same cell indicate significant results in both directions dominate. Question mark indicates that the reviews and meta-analyses did not provide enough data. Gray arrows in CO_2_, warming and CO_2_+warming columns indicate woody tissue responses as specified in reference 2. Small numbers in the upper-right corner of reach cell indicate the source reference: 1 = Koricheva et al., 1998; 2 = Zvereva and Kozlov, 2006; 3 = Valkama et al., 2007; 4 = Peñuelas and Staudt, 2010; 5 = Lindroth, 2012; 6 = Julkunen-Tiitto et al., 2015; 7 = Robinson et al., 2012; 8 = Li et al., 2017. References 1–3 and 7–8 are meta-analyses and 4–6 literature reviews. Meta-analyses give the size of the effect, and the reviews give the frequency of observations in each response categories (–, 0, +).

We summarize the major results of literature reviews and meta-analyses on the quantitative responses of tree PSCs to specific CC-related abiotic factors, with restriction to literature up to 2015 in **Figure 2**. Recent results of PSC (after 2015) and plant VOC (after 2012) research are then reviewed. We then discuss the roles of multiple CC-related stresses on PSCs of forest trees and the potential of non-volatile and volatile PSCs to modulate plant adaptation to CC and their involvement in climate feedback.

## Summary of Older Reviews

Terpenoids and phenolic compounds in leaves and terpenoids in emissions are the most frequently studied PSCs of forest trees in response to CC-related factors (**Figure 2**). Major CC factors such as elevated CO_2_ and warming have the most distinctive, and often contrasting effects on PSCs. Greater availability of CO_2_ increases phenolics in foliage and reduces terpenoids both in foliage and in emissions, while warming reduces phenolics in foliage and increases terpenoids in foliage and emissions (Zvereva and Kozlov, 2006; Peñuelas and Staudt, 2010). CO_2_+warming increased phenolics in foliage, but reduced phenolics in woody tissues of woody plants (Zvereva and Kozlov, 2006). O_3_ effects on foliar concentrations of terpenes and volatile emissions have been variable, but increased phenolic compounds have been found. One reason for variable responses of terpenes could be within-species variation in O_3_ sensitivity (Valkama et al., 2007) and variable O_3_ reactivity of terpenes (Blande et al., 2014). Major terpenoid groups such as monoterpenes (MT) and sesquiterpenes (SQT) in foliage have been less studied separately under most of the other CC related stresses.

## Responses to Abiotic Factors – PSC Concentrations in Trees

Certain plant PSC groups such as flavonoids (Lavola et al., 2013) and MTs (Semiz et al., 2007) are under strong genotypic control. This may increase the potential for forest tree chemical defenses to evolutionarily adapt to environmental changes (Lavola et al., 2013). However, high within-species variation in PSCs is problematic for short-term experimental set-ups and may mask the effects of environmental factors on PSC concentrations and emissions.

### Elevated CO_2_ and Warming

Total leaf phenolics, particularly condensed tannins (Julkunen-Tiitto et al., 2015), follow the traditional carbon-nutrient balance hypothesis (Bryant et al., 1983), which predicts that photosynthesised carbon will be diverted to carbon-rich PSCs, if photosynthesis or CO_2_ availability are at a high level and nutrient availability limits carbon allocation to plant growth (Koricheva et al., 1998). However, many flavonoids and other phenolics, and particularly terpenoids, do not support that hypothesis (Lindroth, 2012). Recent studies have confirmed a trend with foliar phenolics; doubled ambient CO_2_ increased salicylates and phenolic acids (Sobuj et al., 2018), anthocyanins and flavonoids (Vanzo et al., 2015; Nissinen et al., 2016), while less than doubled CO_2_ did not affect phenolics (McKiernan et al., 2012; Randriamanana et al., 2018). Furthermore, some phenolic groups, such as phenolic glycosides, are not as responsive (Jamieson et al., 2017). In *Eucalyptus* spp., there was no observed effect of elevated CO_2_ (McKiernan et al., 2012) or elevated CO_2_ decreased (Bustos-Segura et al., 2017) stored foliar terpenes.

Reduction in foliar phenolics at elevated temperatures (Julkunen-Tiitto et al., 2015) has been explained by a dilution effect whereby more carbon was allocated to growth supporting structures with turnover of phenolics slow or even absent (Kosonen et al., 2012). Recently, it has been reported that warming reduces flavonoids (Nissinen et al., 2016; Zhang et al., 2018), salicylates and phenolic acids (Sivadasan et al., 2018) in deciduous trees and total phenolics in a conifer (Zhang et al., 2018). Flavonoids, alkaloids, and saponins (triterpenes) were increased in *Robinia pseudoacacia* grown in warmer open-top chambers compared to seedlings grown in an open field (Zhao et al., 2016). However, enclosure may significantly affect foliar PSCs (Peltonen et al., 2005).

Warming induced increase in foliar terpenes (Valkama et al., 2007; Julkunen-Tiitto et al., 2015) is possibly explained by improved plant growth and larger storage space for stored terpenes such as MTs and resin acids (Sallas et al., 2003). Recent results showing higher MT concentrations in xylem resin (Ferrenberg et al., 2017) and increased number of needle resin canals (Kivimäenpää et al., 2016) in *Pinus* spp. in warmer environments supports this view. Warming (+2°C) increased concentrations of *Picea abies* needle alkaloids (Virjamo et al., 2014) and parental temperature range correlated with conifer alkaloid concentrations (Gerson et al., 2009; Virjamo and Julkunen-Tiitto, 2016).

### O_3_

O_3_ is phytotoxic and reduces plant photosynthesis and growth (Bueker et al., 2015; Li et al., 2017) and induces substantial changes in protein activity, gene expression, signaling pathways, and metabolism even before any tissue damage can be detected (Bueker et al., 2015; Vainonen and Kangasjärvi, 2015). Activated PSC metabolism (increased biosynthesis and reduced turnover) explains the increased concentrations of phenolic and terpenoid PSCs in tree foliage found in a meta-analysis (Valkama et al., 2007). In response to O_3_ treatment, elevated levels of total phenolics (Gao et al., 2016), condensed tannins (Couture et al., 2017) and flavonoids (Cotrozzi et al., 2018) were found in foliage of deciduous trees, but this was not the case in needles of coniferous trees (Riikonen et al., 2012).

### Drought

Drought in warm periods is expected to become the most frequent widespread climatic extreme that negatively affects terrestrial ecosystems (Schwalm et al., 2017). Forests in southern Europe will face stronger summer drought than those in the north (Ruosteenoja et al., 2018). The observed trends in PSCs are not strong, although reduction of some terpenes and phenolics have been reported (Koricheva et al., 1998; Julkunen-Tiitto et al., 2015). In *Quercus ilex* leaves, moderate drought periods resulted in higher concentrations of total polyphenolics (Nogues et al., 2014; Rivas-Ubach et al., 2014). Drought stress did not affect alkaloid concentrations in conifers (Gerson and Kelsey, 2004). Increasing severity of drought increased resin canal density and resin acid content in the stem wood of *Pinus sylvestris* (Heijari et al., 2010). Contents of most MTs and sesquiterpenoids in needles increased with increasing severity of water deficit, but resin acids showed the opposite trend and polyphenols did not respond (Sancho-Knapik et al., 2017). Drought slightly increased the needle MT contents of the coastal, but not of the interior *Pseudotsuga menziesii* provenance (Kleiber et al., 2017).

### Shading

Shading limits photosynthesis and it is predicted that less carbon is allocated to PSCs in shaded conditions (Koricheva et al., 1998). Giertych et al. (2015) tested the hypothesis with light-demanding and shade-tolerant deciduous tree species. All species had higher total phenolic content of leaves in sunny conditions, but shade tolerant species invested more carbon into growth when more light was available.

### UV-B

Short-wave (280–315 nm) UV-B radiation induces tree foliage to produce more phenolic acids and flavonoids as protective pigments (Julkunen-Tiitto et al., 2015; Kaling et al., 2015). In *Populus tremula* seedlings, two flavonoid compounds increased under enhanced UV-B radiation (Nissinen et al., 2017). Filtering out solar UV-B radiation from reaching *P. tremula* in mountains lead to lower flavonoid concentration, but increased salicin in leaves, and salicylates and total phenolics in stem (Stromme et al., 2018). However, a three-year exposure of *Salix myrsinifolia* to 32% elevated UV-B in a field site did not result in significant changes in flavonoid concentrations, and in male trees total salicylates were even reduced (Ruuhola et al., 2018). Enhanced levels of UV-B radiation did not affect alkaloid concentrations in conifers (Virjamo et al., 2014).

## Responses to Abiotic Factors – Emission of Volatile PSCs

### Warming

Warming is one of the most intensively studied factors in volatile PSC studies (Peñuelas and Staudt, 2010), due to VOC emissions being strongly light and temperature dependent and some, e.g., isoprene not being stored in leaves (Sharkey and Yeh, 2001). At the global scale, isoprene represents nearly 50% of the estimated biogenic VOC emission, and emissions of MTs and SQTs together comprise about 15 and 3%, respectively (Guenther et al., 2012). Warming increased emissions of volatile terpenoids most consistently (Peñuelas and Staudt, 2010) by stimulating biosynthesis (Loreto and Schnitzler, 2010; Fini et al., 2017) and temperature-dependent release from leaf storage structures (Grote and Niinemets, 2008; Peñuelas and Staudt, 2010). VOC emissions from storage structures are high in conifers, e.g., over 40% in *Pinus*, and very low in deciduous trees (Ghirardo et al., 2010). Differences in emission responses of MTs and SQTs to temperature might be caused by temporal storage of less volatile SQTs in leaf surface waxes (Joensuu et al., 2016), and their temperature-dependent adherence and release (Himanen et al., 2015).

Warming of +3°C increased (∼70%) emission rates of isoprene-emitting and MT-emitting *Quercus* spp. (Staudt et al., 2017). Isoprene-emitting *Populus* spp. have an emission maximum at +45°C even if grown in elevated CO_2_ (Potosnak et al., 2014; Niinemets and Sun, 2015). Long-term warming of 1°C+ambient increased MT (fivefold), SQT (fourfold) and green leaf volatile (40%) emissions of *Betula pendula* (Hartikainen et al., 2012). Ontogeny of needle development in terpene-storing conifers affects variability in MT emission rate responses to warming (Esposito et al., 2016; Kivimäenpää et al., 2016; Ghimire et al., 2017; Tiiva et al., 2018). Some specific MTs of forest trees, particularly β-ocimene are known to be heat-stress indicators (Jardine et al., 2017).

### CO_2_

There is clear evidence that exposure to elevated CO_2_ reduces terpenoid-based plant VOC emissions (Peñuelas and Staudt, 2010; Potosnak et al., 2014; Niinemets and Sun, 2015; Mochizuki et al., 2017) and uncouples isoprene emissions from photosynthesis. Inhibition of isoprene synthesis becomes stronger with increasing CO_2_ concentration (Wilkinson et al., 2009; Rasulov et al., 2018) and isoprene is more responsive than MTs (Peñuelas and Staudt, 2010). There is still no consensus of the mechanisms that lead to the contrasting responses of photosynthesis and isoprene biosynthesis (Fini et al., 2017) and emission at elevated CO_2_ (Rasulov et al., 2018). In MT-storing gymnosperms the reduced MT needle concentration at elevated CO_2_ (Sallas et al., 2003) may partly explain reduced MT emissions.

### O_3_

Plant VOCs easily react with O_3_ and samples may be destroyed if ozone scrubbers are not used in sampling (Blande et al., 2014). O_3_ exposure experiments have shown that plant VOC responses are variable and depend on the O_3_ concentration (Peñuelas and Staudt, 2010). Acute ozone exposure can induce similar VOCs to herbivore damage (Blande et al., 2014). Moderately elevated O_3_ concentrations increase emissions of MTs in coniferous (Kivimäenpää et al., 2013, 2016; Ghimire et al., 2017) and deciduous trees (Carriero et al., 2016), but decrease isoprene emission in poplar (Yuan et al., 2016) and MT emissions in *Larix* were not affected (Mochizuki et al., 2017).

### Drought

Warm and dry periods lead to drought stress; mild drought may increase VOC emissions (Peñuelas and Staudt, 2010; Bourtsoukidis et al., 2014; Yuan et al., 2016), not affect (Nogues et al., 2014) or have increasing-decreasing trend (Simpraga et al., 2011), while severe drought results in a reduction of emissions (Peñuelas and Staudt, 2010; Rodriguez-Calcerrada et al., 2013; Luepke et al., 2016; Marino et al., 2017; Saunier et al., 2017; Staudt et al., 2017). Drought stimulated herbivore-induced GLV and MT emissions in *Alnus glutinosa* (Copolovici et al., 2014).

### Shading

Shading by branch position can reduce isoprene and MT emissions from forest trees (Esposito et al., 2016; Juran et al., 2017; van Meeningen et al., 2017). This suggests that cloudiness that reduces penetration of solar radiation to the canopy (Niinemets, 2018) may reduce carbon allocation to volatile PSCs. However, in the canopy of *Fagus sylvatica*, MT emissions were highest in the semi-shaded leaves (Simpraga et al., 2013).

## Combined Abiotic Factors Versus Non-Volatile and Volatile PSCs

The effects of eutrophication [nitrogen (N) deposition and forest fertilization] on tree VOCs are poorly known, although it appears to slightly increase isoprene emissions (**Figure 2**; Peñuelas and Staudt, 2010). Generally, exposure to additional abiotic factors may mitigate, intensify or not affect foliar chemical defense (Sallas et al., 2001; Zvereva and Kozlov, 2006; Vapaavuori et al., 2009) or VOC (Kivimäenpää et al., 2016; Ghimire et al., 2017; Tiiva et al., 2017) responses to the first factor. For example elevated O_3_ and N availability had additive effects on terpenoid emissions from a deciduous tree (Carriero et al., 2016) and conifer (Kivimäenpää et al., 2016), whereas N in addition to warming suppressed the emissions of non-oxygenated MTs from a conifer (Ghimire et al., 2017). In *Populus* sp. N addition (Yuan et al., 2017) or drought (Yuan et al., 2016) did not mitigate the negative effects of O_3_ on isoprene emission.

An elevated CO_2_-related decrease of terpenoids in conifer needles was mitigated by elevated temperature and CO_2_-induced increase in phenolics was intensified by elevated temperature (Zvereva and Kozlov, 2006). Furthermore, elevated CO_2_ may mitigate a typical O_3_ response such as ozone-induced increase of some phenolics in *B. pendula* (Vapaavuori et al., 2009), but in *Larix* CO_2_ and O_3_ did not have interactive effects on volatiles (Mochizuki, et al., 2017). Along altitudinal gradients, the total phenolics in *Quercus robur* foliage were found to decrease at lower elevations where temperature was warmer (Abdala-Roberts et al., 2016). Our analysis shows that in addition to a warming-induced reduction in total phenolics, elevated UV-B radiation may increase flavonoids and phenolic acids in foliage (Julkunen-Tiitto et al., 2015). However, recent combined warming and UV-B exposure outdoors has confirmed that warming has a stronger increasing effect on leaf phenolics (Nissinen et al., 2017) and terpenoid emissions (Maja et al., 2016) than elevated UV-B radiation.

## Plant PSCs and Biotic Stress

Abiotic stresses affect growth, reproduction and behavior of herbivorous insects and severity of microbial plant pathogens and their biotic stress on trees (**Figure 1**). Furthermore, arrival of exotic invasive herbivores and pathogens with warming related to CC may add another threat, because local tree species have not adapted to use PSCs to defend against these invasive species (Donnelly et al., 2012). Increased accumulation of PSCs in plant tissues under elevated atmospheric CO_2_ resulted in compensation feeding and more severe defoliation (Docherty et al., 1996). Warming may reduce constitutive PSCs in foliage (Stark et al., 2015) and improve (Grainger and Gilbert, 2017) or reduce (Ghimire et al., 2017) insect performance and lead to induced production of non-volatile (Rubert-Nason et al., 2015) or volatile PSCs (Blande et al., 2010; Holopainen and Gershenzon, 2010).

## PSC Transmitted Ecosystem Feedbacks to Climate Change

Forests are a globally important sink of atmospheric CO_2_ (Pan et al., 2011; Pukkala, 2018). Carbon is sequestered in soil carbon (44%), living biomass (42%), deadwood (8%), and litter (5%) (Pan et al., 2011). Reducing forest harvesting and adding dead wood as large trees is the fastest way to increase CO_2_ sequestration in boreal forests (Pukkala, 2018). Reduced decay rate of dead wood decreases release of CO_2_ back to the atmosphere. Concentrations of both resin acids (Karppanen et al., 2007) and stilbenes (Ioannidis et al., 2017) in heartwood of *Pinus* sp. can exceed 10% of dry weight and significantly reduce decay rate of dead wood by fungal rot (Karppanen et al., 2007) and by woodborers (Nerg et al., 2004).

The PSCs are released into the soil (Kainulainen et al., 2003) and atmosphere (Aaltonen et al., 2011) faster from the needle litter than dead wood. In conifer forests, MTs contribute over 90% of litter emissions peaking in early summer and during the needle fall in autumn (Aaltonen et al., 2011). After 19-months of decomposition, pine needles have lost 96, 79, and 86 percent of initial concentrations of MTs, resin acids and total phenolics, respectively (Kainulainen et al., 2003). Warm periods in the boreal conifer forests increase reactive MT emissions to the forest atmosphere and stimulate formation of SOA, particularly in evenings (Rose et al., 2018) and potentially stimulate cloudiness (Joutsensaari et al., 2015; Zhao et al., 2017), thereby reducing penetration of solar radiation to vegetation and mitigating warming and drought stress (Niinemets, 2018).

Volatile PSCs have several functions in the species interactions affecting herbivores and their natural enemies and activating defenses in healthy plants (Blande et al., 2014). CC factors also affect herbivore-induced volatiles, but studies on forest trees and woody plants remain limited (Holopainen and Gershenzon, 2010). Non-volatile PSCs released from plant leaf and needle litter accumulate in soil and affect C or net N mineralization in forest soils (e.g., Smolander et al., 2012). Growing three seasons at elevated CO_2_ and O_3_ did not affect concentrations of total phenolics and MTs in *P. sylvestris* needles, but at elevated O_3_ resin acids were increased. However, during decomposition this difference in needle litter was lost (Kainulainen et al., 2003). In leaf litter of *B. pendula* growing at elevated CO_2_ and O_3_ concentrations, several phenolic compounds were elevated, and the growth of juvenile earthworms was reduced (Kasurinen et al., 2007) suggesting that CC reduced quality of food for soil animals. In *Alnus incana* litter, UV-A and UV-B exclusion affected concentrations of phenolic groups variably, whereas in birch litter there were no significant differences in phenolic compounds (Kotilainen et al., 2009).

## Concluding Remarks

There is strong evidence that CO_2_ increases phenolic compounds in foliage, but limits terpenoids in foliage and emissions, while warming decreases phenolic compounds in foliage, but increases terpenoids in foliage and emissions. CO_2_ in combination with other abiotic stresses has more variable effects. As current PSC information is mostly from tree seedlings, long-term experiments covering the whole ontogeny of trees in forest ecosystems are needed to better understand CC effects on PSCs in trees. Assessment of the role of volatile PSCs in sun-screening and non-volatile PSCs in control of decay rate of carbon storage in dead wood will be necessary to improve our knowledge of the capacity of PSCs to mitigate CC. Transgenic trees with silenced or over-expressed genes for key volatile PSCs (Kaling et al., 2015; Vanzo et al., 2015) will be important tools in this task.

## Author Contributions

JH drafted the manuscript. All authors listed have made a substantial, direct and intellectual contribution to the work, and approved it for publication.

## Conflict of Interest Statement

The authors declare that the research was conducted in the absence of any commercial or financial relationships that could be construed as a potential conflict of interest.
